# The functions of the cytoskeleton and associated proteins during mitosis and cytokinesis in plant cells

**DOI:** 10.3389/fpls.2015.00282

**Published:** 2015-04-27

**Authors:** Shanwei Li, Tiantian Sun, Haiyun Ren

**Affiliations:** Key Laboratory of Cell Proliferation and Regulation Biology of Ministry of Education, College of Life Science, Beijing Normal UniversityBeijing, China

**Keywords:** cytoskeleton, preprophase band, spindle, phragmoplast, mitosis, cytokinesis, plant

## Abstract

In higher plants, microtubule (MT)-based, and actin filament (AF)-based structures play important roles in mitosis and cytokinesis. Besides the mitotic spindle, the evolution of a band comprising cortical MTs and AFs, namely, the preprophase band (PPB), is evident in plant cells. This band forecasts a specific division plane before the initiation of mitosis. During cytokinesis, another plant-specific cytoskeletal structure called the phragmoplast guides vesicles in the creation of a new cell wall. In addition, a number of cytoskeleton-associated proteins are reportedly involved in the formation and function of the PPB, mitotic spindle, and phragmoplast. This review summarizes current knowledge on the cytoskeleton-associated proteins that mediate the cytoskeletal arrays during mitosis and cytokinesis in plant cells and discusses the interaction between MTs and AFs involved in mitosis and cytokinesis.

## Introduction

In plants, microtubules (MTs) and actin filaments (AFs) are essential components of the basic machineries required for cell division and expansion. Unlike animal cells, plant cells are enfolded in pecto-cellulosic cell walls and do not migrate. Therefore, orientation of the cell division plane is crucial for the cellular organization of plant tissues. The MTs and AFs are involved in the selection of the division plane in preprophase cells and in the formation of the cell plate during cytokinesis (Muller et al. 2009; Liu et al., 2011b; Rasmussen et al., 2013).

The preprophase band (PPB) is a transient ring of cortical MTs and AFs specific to plant cells; PPB delineates the plane of cell division at the onset of mitosis and plays an essential role in division plane specification (Mineyuki, 1999). The PPB appears in the cell cortex in late G2 phase and persists throughout prophase, but disassembles with the breakdown of the nuclear envelope when the mitotic spindle forms (Dixit and Cyr, 2002). Mitotic spindle is a bipolar array of MTs that segregates chromosomes between daughter cells during mitosis. Moreover, an AF cage surrounds the spindle and maintains spindle position during mitosis (Lloyd and Traas, 1988; Katsuta et al., 1990). During cytokinesis, the formation of a new cell plate is accomplished by a dynamic AF- and MT-based structure known as the phragmoplast. The phragmoplast assembles at the center of the cell and grows centrifugally toward the parental cell wall. When the phragmoplast reaches the cortical site formerly marked by the PPB, the cell plate, and parental membranes fuse, thereby completing cytokinesis (Smith, 2001; Van Damme et al., 2007).

Both MT and AF structures play essential roles in cell division because the cell plate does not form in the absence of MTs (Clayton and Lloyd, 1985; Kakimoto and Shibaoka, 1987), and the treatment with actin polymerization inhibitors results in oblique cell plate formation (Hoshino et al., 2003; Sano et al., 2005). Moreover, interactions and cross-talk between MTs and AFs are involved in plant cell division (Wasteneys and Galway, 2003). In this review, we summarize the current findings on the cytoskeleton-associated proteins that mediate the cytoskeletal arrays during mitosis and cytokinesis in plant cells and focus on the MT and AF interactions involved in mitosis and cytokinesis.

## PPB Formation

The PPB is a temporal structure that forms before mitosis. Although some plant species and cell types can divide in the absence of PPBs, e.g., starchy endosperm, meiocytes, and some cultured suspension cells (Otegui and Staehelin, 2000; Chan et al., 2005; Sabelli and Larkins, 2009), pharmacological or genetic disruption of PPBs can cause divisions in aberrant orientations in plant cells that can form PPBs normally (Vanstraelen et al., 2006). These observations suggest that PPB plays a key role in determination of the division plane.

A variety of MT-associated proteins (MAPs) have been identified to participate in PPB formation. *Arabidopsis* MT organization 1 (MOR1), a plant homolog of animal XMAP215, can accelerate both the growth and shrinkage rates of MTs *in vitro* and* in vivo* (Brouhard et al., 2008; Kawamura and Wasteneys, 2008). The MOR1 localizes to PPBs and other MT arrays (Kawamura et al., 2006). In the case of *Arabidopsis thaliana mor1* mutant, nearly one-half of the dividing cells failed to form PPB before spindle formation and those that formed PPB were often disrupted (Kawamura et al., 2006). Tobacco MT-binding protein 200 (TMBP200), a homolog of MOR1, is also found on PPB (Hamada et al., 2004). *Arabidopsis* CLIP-associated protein (CLASP), which shares structural similarity with the XMAP215 family of proteins in animals, is implicated in PPB formation (Mimori-Kiyosue et al., 2005). In *clasp* mutants, PPB tends to be disoriented, and PPB narrowing is retarded compared with wild-type plants (Ambrose et al., 2007). The SABRE protein, which shares similarity with proteins of unknown function in eukaryotes, plays important roles in orientation of cell division and planar polarity. Moreover, *Arabidopsis* SABRE has recently been reported to stabilize the orientation of CLASP-labeled MT in the PPB, which is essential for cell division plane orientation (Pietra et al., 2013). MAP65 is an MT-binding protein family that is involved in PPB formation. MAP65s bundle MTs by forming cross bridges between overlapping MTs, thereby potentially contributing to the stability of PPB MTs via bundling (Smertenko et al., 2004). Katanin is an evolutionarily conserved protein complex for severing MT. In certain root cells of the *lue1* mutant, early PPBs are disorganized and are sustained longer in the prophase stage than wild-type PPBs (Panteris et al., 2011).

Although plant cells lack centrosomes, plant proteins with similarity to the human centrosomal proteins are required for PPB formation. In *A. thaliana*, the TONNEAU1 (TON1) proteins related to the human centrosomal proteins FOP co-localize with PPBs. The *ton1* mutants are unable to form a PPB in *A. thaliana* (Azimzadeh et al., 2008). TON1 Recruiting Motif proteins (TRMs) have been recently shown to interact with TON1 in *Arabidopsis*. One of TRMs (TRM1) is found to bind and recruit TON1 to the cortical MTs (Drevensek et al., 2012). Recently, it has been reported that the activity of a regulatory complex composed of TON1, TRM, and a putative protein phosphatase 2A (PP2A) holoenzyme (TTP) is required for PPB formation and spatial control of cell division. All members of the TTP complex share similarity with animal centrosomal proteins, revealing an evolutionary link between MT organizing mechanisms in plant and other eukaryotes (Spinner et al., 2013).

The actin PPB is considered to be wider than the MT PPB (Palevitz, 1987). The formation of actin PPB depends on MTs because application of MT-depolymerizing drugs prevents formation of both the MT and actin components of the PPB (Palevitz, 1987; Vanstraelen et al., 2006). The actin PPB can also affect the MT PPB because actin depolymerization results in dramatic broadening of the MT PPB and shifting of division planes in dividing cells during the preprophase and prophase stages (Mineyuki and Palevitz, 1990). Thus, AFs and MTs may play indispensable roles in PPB in a coordinated manner. Until recently, some proteins were reported to regulate the cooperation or interaction between AFs and MTs in PPB. *Arabidopsis* formin 14 (AFH14), a type II formin, is found on the PPB and directly binds and bundles AFs and MTs *in vitro* (Li et al., 2010). Moreover, in the presence of both MTs and AFs, AFH14 has higher affinity to MT and preferentially binds to MTs; however, the presence of excessive AFH14 promotes cross-linkages of MTs and AFs (Li et al., 2010). AtKinG, a kinesin 14-type molecular motor from *Arabidopsis*, localizes to MTs and AFs by fluorescence double-labeling; AtKinG strongly labels the PPB in time-lapse cell division studies (Buschmann et al., 2011). NtKCH, a KCH homolog from tobacco BY-2 cells, is suspected to act as an MT–AF cross-linker. In dividing cells, NtKCH accumulates in the PPB (Klotz and Nick, 2012). These results show that different kinds of proteins mediate MTs, AFs, or both MTs and AFs in the PPB, thereby indicating a variety of interactions between AFs and MTs, which participate in PPB formation.

## Spindle Formation and Position

Unlike in animal cells, the mitotic spindle in plant cells originates from the nuclear envelope in prophase (De Mey et al., 1982; Ambrose and Cyr, 2008). Spindle assembly starts prior to PPB breakdown at prometaphase, with the spindle axis perpendicular to the plane of the PPB (Chan et al., 2005; Yoneda et al., 2005). Previous studies have demonstrated that PPB plays a role in the timely formation of a normal bipolar spindle (Ambrose and Cyr, 2008).

Similar to PPB, a number of MAPs have also been shown to participate in spindle formation. Apart from PPB, MOR1/TMBP200, CLASP, and MAP65 are implicated in spindle formation (Kawamura et al., 2006; Ambrose et al., 2007; Fache et al., 2010; Yasuhara and Oe, 2011). Kinesins have been implicated in spindle organization. *A. thaliana* kinesin-related protein 125c (AtKRP125c), a member of the plus end kinesin-5 group, plays a role in establishing the spindle structure and cross-linking antiparallel MTs at the midzone (Wiedemeier et al., 2002; Bannigan et al., 2007). γ-Tubulin is distributed throughout the mitotic spindle and plays an indispensable role in the assembly of the bipolar spindle (Binarova et al., 2006; Pastuglia et al., 2006). *A. thaliana* NEDD1, which acts as an anchoring factor of γ-tubulin complex, decorates spindle MTs preferentially toward theirs minus ends. In *nedd1* mutants, nearly half of the dividing microspores show aberrant MT organization and abnormal spindles, thereby demonstrating the important role of NEDD1 in spindle formation (Zeng et al., 2009). Additionally, repressed γ-tubulin Complex Protein 4 (GCP4) expression by an artificial microRNA results in abnormal spindles in *A. thaliana* (Kong et al., 2010). GCP3-Interacting Protein 1 (GIP1) and GIP2 have been shown to co-localize with γ-tubulin, GCP3, and/or GCP4. In *A. thaliana*, reduced spindle robustness associated with lower amounts of γ-tubulin, GCP3, and GCP4 appears in the *gip1 gip2* double mutants (Janski et al., 2012), implying that all these proteins may function together.

Many studies have shown that the AF cage surrounds the spindle and connects it to the cell periphery, thereby maintaining the spindle’s position during mitosis (Lloyd and Traas, 1988; Katsuta et al., 1990). Recent work shows that disruption of the actin network results in misoriented spindle and oblique cell plates (Kojo et al., 2013). A number of proteins reportedly regulate the interaction between AFs and MTs involved in spindle formation and position. MAP190 from tobacco BY-2 cells co-sediments with both AFs and MTs *in vitro.* Immunocytochemical studies revealed that MAP190 is localized in the spindle (Igarashi et al., 2000). AFH14 is also localized to spindles. T-DNA insertion mutants of AFH14 show MT abnormalities during pollen gametogenesis (Li et al., 2010). Cells overexpressing AFH14 under the control of an inducible promoter increases the resistance to both MT- and AF-depolymerizing drugs, whereas AFH14 loss-of-function causes alterations in MT structures and AF instability (Li et al., 2010). In *Arabidopsis* and BY-2 cells, AFs form a cage around spindle. Interestingly, in AFH14-overexpressing cells, the AFs and MTs co-localize in spindles (Li et al., 2010). These results suggest that proteins that interact with MTs and AFs link spindle MTs to the surrounding actin cage to regulate the spindle formation and position during mitosis.

## The Phragmoplast Establishment and Configuration

Phragmoplast consists of MTs and AFs with their plus ends pointing toward the phragmoplast midzone. The phragmoplast is highly dynamic and expands toward the cell cortex to allow the cell plate growing within it to expand centrifugally. The expansion is a result of continuous MT and AF assembly at the leading edge of phragmoplast while the MTs and AFs toward the center of the phragmoplast are disassembled (Liu et al., 2011a).

A growing number of MAPs and other MT-interacting factors are associated with the phragmoplast are required for the operation of the phragmoplast (Hamada, 2007; Guo et al., 2009). In addition to their involvement in the formation and function of the PPB and/or spindle, MOR1, CLASP, MAP65, AtKRP125c, NEDD1, and GCPs also contribute to phragmoplast establishment and configuration (Muller et al., 2004; Kawamura et al., 2006; Ambrose et al., 2007; Bannigan et al., 2007; Zeng et al., 2009; Kong et al., 2010). PAKRP1 and PAKRP1L in the kinesin-12 family show high homology, and both of which localize to the midzone of the phragmoplast. Mutations at either PAKRP1 or PAKRP1L do not cause a noticeable defect. However, the phragmoplast fails to assemble normally and causes defective cell plate formation in the absence of both kinesins, thereby indicating their redundant function in the phragmoplast (Lee et al., 2007). These two kinesins are assumed to play roles in phragmoplast formation by precluding the plus ends of the opposing MT sets from crossing the midzone (Zhu and Dixit, 2012). Moreover, in the moss *Physcomitrella patens*, MT interdigitation in the phragmoplast depends on the kinesin KINID1, which may function as a motor for vesicle transport in the phragmoplast (Hiwatashi et al., 2008). Recently, KINID1 kinesins have also been shown to play an essential role in organizing MTs during tip growth (Hiwatashi et al., 2014). Consequently, these proteins likely contribute to the maintenance of the bipolar figure of MTs in phragmoplasts by promoting MT polymerization and/or stability. Further studies are needed to clarify the spatio-temporal and functional relationships among these proteins, which are involved in the phragmoplast.

In plant cells, γ-tubulin ring complexes (γ-TuRCs) are capable of initiating MT nucleation at the sides of extant MTs (Murata et al., 2005). However, γ-TuRC fails to interact with the MT array directly. Consequently, protein(s) that directly interact with MTs must mediate the association of γ-TuRC with phragmoplast MTs. Plant augmin complex subunits are required for γ-tubulin recruitment, MT organization in phragmoplast, and cell plate formation (Zeng et al., 2009; Nakamura et al., 2010; Ho et al., 2011; Hotta et al., 2012). The presence of γ-tubulin in the phragmoplast MTs mainly depends on augmin because the mutation causes delocalization of γ-tubulin in the phragmoplast in augmin mutant cells of *A. thaliana* (Hotta et al., 2012). In addition, the augmin mutant phragmoplast MT array often fails to expand centrifugally, and MT bundles become disorganized (Hotta et al., 2012). In the moss *P. patens*, MT formation in phragmoplasts is severely compromised after knockdown of an augmin subunit, thereby leading to incomplete expansion of phragmoplasts (Nakaoka et al., 2012). Thus, MT-dependent MT nucleation mediated by augmin and γ-TuRC may play an important role in the organization of phragmoplast MTs.

Compared with MTs, the role of actin in the phragmoplast is less clear. Tobacco BY-2 cells treated with AF-depolymerizing drugs show disorganized phragmoplasts and wrinkled cell plates (Hoshino et al., 2003; Yoneda et al., 2004; Sano et al., 2005; Higaki et al., 2008; Kojo et al., 2013). Because arrays of AFs and MTs closely co-exist and play important roles in the phragmoplast (Smith, 1999; Yokota et al., 2009), cooperation or interaction between AFs and MTs is assumed, and several proteins have been proposed to mediate the cooperation or interaction between AFs and MTs in the cytoskeletal structure. Cotton kinesin GhKCH2, which decorates the midzone of the phragmoplast in dividing root tip cells, binds to AFs and MTs (Xu et al., 2009). AFH14 has been shown to localize to the phragmoplast (Li et al., 2010). Experiments in both *Arabidopsis* and BY-2 cells show that the length of phragmoplast MTs is longer than that of AFs. However, in AFH14-overexpressing cells, the MTs and AFs appear to be similar in length and are aligned evenly with one another (Li et al., 2010). In addition to localizing to MTs and to AFs, MAP190, AtKinG, and NtKCH also localize to the phragmoplast (Igarashi et al., 2000; Buschmann et al., 2011; Klotz and Nick, 2012). Recently, in moss and tobacco, myosin VIII links phragmoplast MTs to the cortical division site via AFs during phragmoplast expansion; AFs may interact with the MTs bridging the cell cortex and the phragmoplast (Wu and Bezanilla, 2014).

## Conclusion and Perspectives

Plant mitosis and cytokinesis depend on cytoskeletal dynamics. Numerous cytoskeleton-associated proteins involved in mitosis and cytokinesis have already been identified (**Table 1**). Based on the data in **Table 1**, more MAPs than actin-binding proteins have been found during the process of cell division. This may not imply that the role of AFs is less important than that of MTs in cell division. Because of the differences in techniques used for visualization, the sensitivity of AF to fixation, and preservation difficulty, AF observation and imaging seem to be more difficult compared with MT observation and imaging (Lloyd, 1988; Liu and Palevitz, 1992). Technological advances in microscopy imaging will facilitate the observation of AFs involved in cell division. Recent studies using confocal scanning microscopy have shown that cortical AFs are strongly correlated with mitotic spindle and phragmoplast orientations (Kojo et al., 2013, 2014). In addition, MTs and AFs are often co-distributed in the apparatus of cell division, thereby indicating that MTs and AFs may cooperate in a spatially and temporally coordinated manner through specific bifunctional proteins or multiprotein complexes. Over the past years, a growing number of proteins or protein complexes that bridge these cytoskeletal systems have been identified, including the following: MAP190, MAP18, GhKCH1, GhKCH2, OsKCH1, NtKCH, SB401, EB1, and AFH14. The mechanism underlying MT and AF cooperation or interaction remains ambiguous. To elucidate the mechanisms of MT and AF interaction and the regulation of these interactions, the precise function of these cross-linking proteins found and other new proteins involved in the interactions needs to be clarified and identified via proteomics and creative genetic strategies. Furthermore, technological advances in real-time imaging, such as the application of spinning disk confocal microscopy and TIRF microscopy, will potentially push forward the investigation of this issue.

**Table 1:** Cytoskeleton-associated proteins involved in plant mitosis and/or cytokinesis.

Cytoskeleton-associated proteins	Proteins	Location	Function	Reference
MT-associated proteins (MAPs)	MOR1/TMBP200	PPB; spindle and phragmoplast	Organize/stabilize MTs	Hamada et al. (2004), Kawamura et al. (2006), Kawamura and Wasteneys (2008)
	CLASP	PPB; spindle and phragmoplast	Bind to MT plus end	Ambrose et al. (2007)
	SABRE	Plasma membrane, endomembranes, spindle, and cell plate	Stabilize CLASP-labeled PPB MTs	Pietra et al. (2013)
	MAP65	PPB and phragmoplast	Bundle MTs	Muller et al. (2004), Smertenko et al. (2004)
	Lue1	Cortical MTs	Sever MT	Panteris et al. (2011)
	TON1	PPB MTs	Organize/stabilize PPB MTs	Azimzadeh et al. (2008), Spinner et al. (2013)
	TRM1	Cortical MTs	Target TON1 to cortical MT	Drevensek et al. (2012), Spinner et al. (2013)
	AFH14	PPB; spindle and phragmoplast	Bundle MTs and actin filaments (AFs) and cross-link them	Li et al. (2010)
	AtKinG	PPB and phragmoplast	Minus-end directed kinesin	Buschmann et al. (2011)
	NtKCH	PPB and phragmoplast	Associate with both MTs and AFs	Klotz and Nick (2012)
	AtKRP125c	PPB; spindle and phragmoplast	Plus-end directed kinesin	Bannigan et al. (2007)
	γ-tubulin	Spindle and phragmoplast	Nucleate/organize MTs	Binarova et al. (2006), Pastuglia et al. (2006)
	NEDD1	Spindle and phragmoplast	Nucleate/organize MTs	Zeng et al. (2009)
	GCP3	Nuclear envelope	Required for nuclear envelope-based MT nucleation	Nakamura and Hashimoto (2009)
	GCP4	Spindle and phragmoplast	Nucleate/organize MTs and facilitate interaction between γ-tubulin and MTs	Kong et al. (2010)
	GIP1, GIP2	PPB; spindle and phragmoplast	Organize MTs	Janski et al. (2012)
	MAP190	Spindle and phragmoplast	Bind to and bundle MTs and AFs	Igarashi et al. (2000)
	PAKRP1, PAKRP1L	Phragmoplast	Plus-end directed kinesin	Lee et al. (2007)
	KINID1	Phragmoplast	Kinesin for interdigitated MTs	Hiwatashi et al. (2008)
	γ-TuRC	Spindle and phragmoplast	Nucleate/organize MTs	Murata et al. (2005), Zeng et al. (2009)
	Augmin	Spindle and phragmoplast	Activate γ-TuRC	Nakaoka et al. (2012)
	GhKCH2	Phragmoplast and cell plate	Bundle MTs and AFs and cross-link them	Xu et al. (2009)
Actin-associated proteins	AFH14	PPB; spindle and phragmoplast	Bundle MTs and AFs and cross-link them	Li et al. (2010)
	AtKinG	PPB and phragmoplast	Minus-end directed kinesin	Buschmann et al. (2011)
	NtKCH	PPB and phragmoplast	Associate with both MTS and AFs	Klotz and Nick (2012)
	MAP190	Spindle and phragmoplast	Bind to and bundle MTs and AFs	Igarashi et al. (2000)
	GhKCH2	Phragmoplast and cell plate	Bundle MTs and AFs and cross-link them	Xu et al. (2009)
	Myosin VIII	Spindle and phragmoplast	Actin-based molecular motors	Wu and Bezanilla (2014)

## Conflict of Interest Statement

The authors declare that the research was conducted in the absence of any commercial or financial relationships that could be construed as a potential conflict of interest.
